# The Use of Abatacept for the Treatment of Felty Syndrome in Rheumatoid Arthritis

**DOI:** 10.7759/cureus.46086

**Published:** 2023-09-27

**Authors:** Rishika V Chin, Sheila Serin, Ahmed Khan, Keneisha Smith, Suresh Kumar

**Affiliations:** 1 Internal Medicine, University of Miami John F. Kennedy (JFK) Medical Center, Atlantis, USA; 2 Rheumatology, West Palm Veterans Affairs Medical Center, West Palm Beach, USA

**Keywords:** severe neutropenia, “pancytopenia”, rhuematoid arthritis, felty’s syndrome, abatacept

## Abstract

Felty syndrome is characterized by a triad of rheumatoid arthritis, neutropenia, and splenomegaly that typically occurs in patients with seropositive rheumatoid arthritis. We report a case of an 81-year-old man with a 22-year history of rheumatoid arthritis who developed pancytopenia and neutropenia for one month. An abdominal ultrasound revealed splenomegaly. A bone marrow biopsy excluded large granular lymphocytic leukemia, thus Felty Syndrome was diagnosed. He was treated with a maximum tolerated dose of methotrexate (15 mg once a week) for two months with no improvement in absolute neutrophil count. The patient was given abatacept 1000 mg intravenously every four weeks with improvement in hemoglobin, leukocyte, and platelet counts and absolute neutrophil counts from 0.2 to 2.4 K/µl.

## Introduction

Felty syndrome comprises a triad of rheumatoid arthritis, neutropenia, and splenomegaly that usually occurs in patients with long-standing, seropositive disease with destructive joint involvement. Its prevalence is estimated to be 1-3% of patients with rheumatoid arthritis [1]. It was first described by Augustus Felty in 1924 at the Johns Hopkins Hospital when he described five cases of patients with chronic arthritis of about four years duration with splenomegaly and leukopenia [2].

The diagnosis of Felty syndrome does not require the entire triad, but neutropenia with an absolute neutrophil count, generally less than 2000/mm^3^, is required. There are no randomized control studies describing the management of Felty syndrome, but the first line of treatment is considered to be methotrexate [2], with other treatments described in case reports. Here, we describe the case of an 81-year-old man with a history of seropositive rheumatoid arthritis who presented with worsening pancytopenia and was treated successfully with abatacept.

## Case presentation

An 81-year-old man with a history of seropositive rheumatoid arthritis (positive rheumatoid factor and anti-cyclic citrullinated peptide antibody) diagnosed 22 years ago, left knee osteoarthritis with a history of left knee arthroplasty with prosthetic joint infections, and chronic kidney disease stage three B presented to a rheumatology clinic with worsening pancytopenia for one month.

He had not been maintained on disease-modifying anti-rheumatic drug (DMARD) therapy for six months. After being diagnosed with rheumatoid arthritis, he was started on methotrexate and infliximab for approximately one year. He had a left knee replacement, which was complicated by a joint infection, and infliximab was subsequently discontinued. He was then placed on leflunomide, which was discontinued due to pruritus and persistent diarrhea. Subsequently, he was started on sulfasalazine, which was discontinued due to gastrointestinal side effects. He continued methotrexate (15 mg) and was placed on etanercept 50 mg subcutaneously daily. Etanercept was discontinued seven years after diagnosis due to a persistent left knee hardware infection. Methotrexate was also discontinued due to elevated liver function tests. After removal of the infected left knee hardware and treatment with antibiotics, etanercept 50 mg subcutaneously daily was resumed. The patient then discontinued etanercept himself six months prior to developing pancytopenia.

A physical examination revealed no synovitis in the wrists, hands, or feet bilaterally. Cardiac and respiratory examinations revealed no abnormalities.

His complete blood count revealed pancytopenia with a low absolute neutrophil count, as displayed in Table 1. His ESR was 7 mm/hour, and hepatitis B and C titers were negative. Anti-cyclic citrullinated peptide (CCP) antibody titers were greater than 250 units. An abdominal ultrasound was performed and revealed splenomegaly with a length of 17.4 cm. He underwent a bone marrow biopsy, which showed immunophenotypic evidence of left-shifted maturation and no evidence of increased blasts or clonal lymphoid expansion. There was no evidence of T-cell lymphoproliferative disorder by flow cytometry, thus ruling out large granular lymphocytic (LGL) leukemia. Due to the absence of LGL leukemia, Felty syndrome was diagnosed.

**Table 1:** Complete blood count and absolute neutrophil count before and after treatment with abatacept

Test	Results prior to treatment	Results 4 days after 1^st^ treatment	Results 1 month after 1^st^ treatment	Reference range	Units
Hemoglobin	11.6	12.0	14.8	13.4–17.5	g/dL
White blood cell count	1.5	2.2	3.9	4.0–11.0	K/µL
Absolute neutrophil count	0.5	0.6	2.4	1.8–7.8	K/µL
Platelet count	103	158	142	140–400	K/µL

He was prescribed methotrexate 7.5 mg orally once a week, then increased to 15 mg once a week for two months. Due to persistent pancytopenia with no improvement in neutrophil count, he was started on Abatacept 1000 mg intravenously every four weeks. His white blood cell, absolute neutrophil, and platelet count increased significantly within four days and one month after treatment, as displayed in Table 1.

## Discussion

Felty syndrome comprises a triad of rheumatoid arthritis, neutropenia, and splenomegaly that usually occurs in patients with long-standing, seropositive disease with destructive joint involvement. Its prevalence is estimated to be 1-3% of patients with rheumatoid arthritis [1]. The goal of treatment is to control symptoms of rheumatoid arthritis and to achieve a granulocyte count above 2000 cells per microliter to prevent infections [2]. The exact pathophysiology of Felty syndrome is unknown but likely multifactorial. Proposed mechanisms include immune-related destruction of neutrophils, increased splenic sequestration of neutrophils, and inadequate production of neutrophils due to bone marrow infiltration by cytotoxic lymphocytes [2]. In a case-control study performed by Hellmich et al. [3], nine out of 15 patients with Felty syndrome were found to have higher serum G-CSF than patients with rheumatoid arthritis; nine patients had anti-G-CSF autoantibodies, which were higher in patients with neutropenia.

The treatment of Felty syndrome has been mainly obtained from observational studies. The preferred first-line treatment is generally methotrexate, which has been shown to have an improvement in granulocyte count after four weeks of treatment in several studies [4-5]. This patient was placed on methotrexate for eight weeks with no improvement in leukocyte or neutrophil count.

Leflunomide has also been used for the treatment of Felty syndrome in a case report with improvements in white blood cells and absolute neutrophil counts [6]. This was not considered an option for this patient due to his previous adverse reactions and gastrointestinal side effects.

Granulocyte colony-stimulating factor (G-CSF) has been used in patients with Felty syndrome with an increase in the absolute neutrophil count within one week of treatment. However, long-term G-CSF therapy has a risk of exacerbating the underlying autoimmune condition and a decrease in the absolute neutrophil count after discontinuation of the medication, but with stabilization at a level above the pretreatment count [7].

Of significance, a systematic review of biological therapies in Felty syndrome, including etanercept, infliximab, and adalimumab, has not been shown to be effective in treatment [8]. Rituximab has been considered the second-line treatment for Felty syndrome with improvement in neutrophil count in two case reports [9-10]. He developed pancytopenia over six months after self-discontinuing etanercept, and it was not resumed due to its poor efficacy in the treatment of Felty syndrome.

Abatacept is a recombinant fusion protein comprising the extracellular domain of human CTLA4 and a fragment of the Fc domain of human IgG1, which has been modified to prevent complement fixation. It competes with CD28 for CD80 and CD86 binding on T cells and selectively modulates the costimulatory signal required for full T-cell activation [11]. Several clinical studies have shown the effectiveness and safety profile of abatacept in rheumatoid arthritis [11], but there is a paucity of data describing its effectiveness in Felty syndrome. Two case reports have shown effective treatment of Felty syndrome thus far, with improvements in neutropenia [12-13]. Abatacept has been found to decrease the proportion of T follicular helper cells, which are critical for the formation and function of B cells, which are integral to the pathogenesis of autoimmune diseases. It also significantly reduced the titers of both rheumatoid factor and anti-cyclic citrullinated peptide antibody levels [14].

In this case, the patient was treated with methotrexate for eight weeks, with no improvement in neutropenia or his leukocyte count. He had intolerance of previous DMARDs and was subsequently placed on abatacept. He had significant improvement in neutropenia, platelet count, and hemoglobin after two months of treatment, with a sustained increase one month after treatment and no reported adverse reactions.

## Conclusions

There is no consensus on the treatment of Felty syndrome, and there is a paucity of data regarding the use of abatacept in improving granulocyte count. This case demonstrates an improvement in leukocyte count, absolute neutrophil count, platelet count, and hemoglobin with the use of abatacept. In conclusion, further prospective studies should be done to investigate the use of abatacept as an alternative second-line agent for the treatment of pancytopenia in Felty syndrome.
